# Spin and Momentum Mapping of Highly Oriented Spinterfaces

**DOI:** 10.1021/acs.nanolett.5c04710

**Published:** 2025-11-28

**Authors:** Iulia Cojocariu, Daniel Baranowski, Vitaliy Feyer, Matteo Jugovac, Claus Michael Schneider

**Affiliations:** ◪ Physics Department, 9315 University of Trieste, 34127 Trieste, Italy; ‡ 18474 Elettra Sincrotrone Trieste S.C.p.A., 34149 Basovizza Trieste, Italy; § 28334 Peter Grünberg Institute (PGI-6), Forschungszentrum Jülich GmbH, 52425 Jülich, Germany; ∥ Faculty of Physics and Center for Nanointegration Duisburg-Essen (CENIDE), University of Duisburg-Essen, 47048 Duisburg, Germany; ⊥ Department of Physics and Astronomy, UC Davis, Davis, California 95616, United States

**Keywords:** Spinterfaces, Scattering, Spin polarization, Hybrid interfaces

## Abstract

Structurally defined interfaces between magnetic substrates and molecular layers, so-called *spinterfaces*, represent a critical frontier in the design of spin-functional devices. Here, we show that long-range molecular order enables coherent Umklapp scattering of spin-polarized substrate electrons, modifying the spin-resolved electronic structure at the interface. Using spin-resolved momentum microscopy and photoemission tomography, we compare iron (FePc) and metal-free phthalocyanine (H_2_Pc) monolayers assembled on an oxygen-passivated iron surface. We find that both molecular lattices give rise to distinct Umklapp replicas of the substrate valence bands. By selectively probing the momentum space, we demonstrate that spin polarization near normal emission is predominantly governed by scattering rather than direct contributions from possibly spin-polarized molecular orbitals. This work underscores the intricate relationship between structural order and spin functionality, providing valuable insights into engineering spinterfaces.

## Graphical abstract

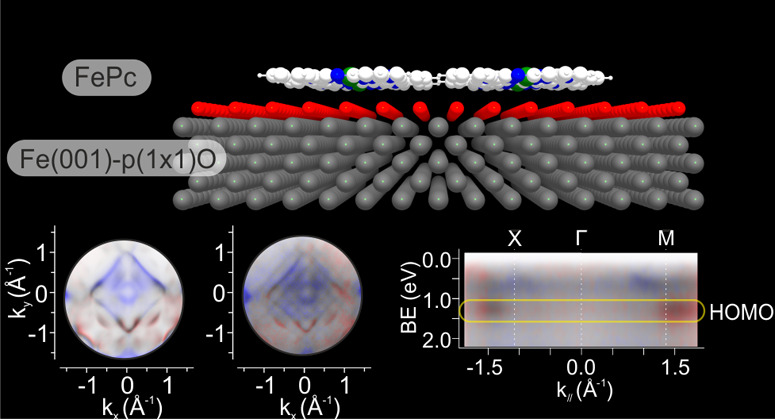

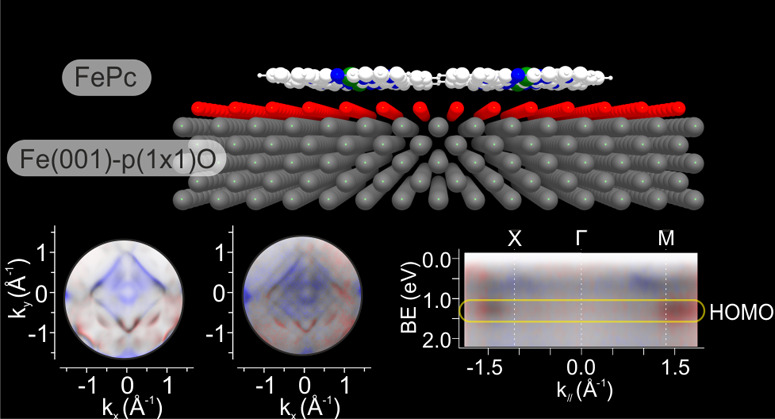

Organic–ferromagnetic interfaces, known as *spinterfaces*, are rapidly emerging as key enablers of next-generation spintronic devices. These hybrid systems combine the spin functionality of magnetic substrates with the tunability and nanoscale precision of molecular layers, offering potential for low-power, flexible, and molecularly customizable spin-based logic and memory.^1–3^ Despite this promise, achieving robust and controllable spin polarization at such interfaces remains a major challenge, largely due to complex interfacial interactions and structural disorder on the molecular scale.

Traditional strategies for engineering spinterfaces have focused on direct spin hybridization or exchange coupling between molecular orbitals and magnetic substrates. Such approaches have demonstrated that transition-metal porphyrins and phthalocyanines (TM-P and TM-Pc) can stabilize spin-polarized states via coupling with ferromagnetic films, enabling magnetic order even at room temperature.^4,5^ Modulation of this coupling, for instance by introducing atomic interlayers, allows transitions between ferromagnetic and antiferromagnetic coupling regimes, offering the ability to finely tune spinterface properties.^6–9^ Chemisorption of these molecules can facilitate hybridization between their π-orbitals and substrate electronic states, resulting in a modified interfacial electronic structure with enhanced magnetic stability.^10,11^ This hybridization not only amplifies the substrate’s magnetic response but can also lead to the formation of hybrid interface states (HISs) near the Fermi level, which significantly impact spin-injection efficiency.^12,13^ HISs with strong spin polarization have been observed in specific combinations of metal-phthalocyanines and ferromagnetic surfaces.^14–17^

Despite this growing understanding, a key challenge remains: disentangling the role of structural order from genuine electronic hybridization in governing spin polarization at organic–ferromagnetic interfaces. Long-range periodicity in molecular overlayers can introduce reciprocal lattice vectors that scatter substrate-derived photoelectrons, giving rise to momentum-resolved signatures, such as Umklapp replicas, that mimic or obscure the electronic states of the interface.^18–22^ Yet, the extent to which such elastic scattering processes influence spin-resolved signals remains largely underexplored. Distinguishing between spin-polarized hybrid states and spin-conserving diffraction replicas is essential for correctly interpreting spectroscopic data and designing spinterfaces with predictable spin functionality.

In this work, we explore well-ordered spinterfaces based on iron- and metal-free phthalocyanines (FePc and H_2_Pc) assembled on an oxygen-passivated Fe(001)-p(1 × 1)O substrate, a magnetically active, chemically stable platform that enables the formation of a highly ordered molecular superstructure. By comparing FePc and H_2_Pc under identical conditions, we isolate the influence of the central metal ion from coherent scattering effects arising from the periodic molecular lattice. Spin-resolved momentum microscopy combined with photoemission tomography reveals that even in the absence of a magnetic center the ordered overlayer redistributes the substrate spin-polarized bands via Umklapp scattering. These results highlight the critical role of interfacial symmetry and long-range molecular order in governing the detected electronic and spin-resolved properties of spinterfaces.

Engineering spinterfaces requires careful control over interfacial interactions as the direct deposition of organic molecules onto metals often leads to substantial modifications of their structural and electronic properties.^23,24^ One strategy to modulate such interactions involves the introduction of ultrathin metal oxide layers, which act as a buffer by tuning the interfacial charge transfer and orbital hybridization.^25,26^ In this context, the Fe(001)-p(1 × 1)O surface serves as a particularly suitable substrate. The oxygen monolayer passivates the reactive iron surface while preserving its intrinsic ferromagnetism, offering a well-defined and magnetically active template for molecular adsorption. Importantly, its periodic atomic structure facilitates the self-assembly of planar organic molecules, such as phthalocyanines, into ordered overlayers.^26,27^

Two model systems, consisting of monolayer FePc and H_2_Pc molecules deposited on Fe(001)-p(1 × 1)­O, are investigated herein. This comparative approach enables us to isolate the influence of the central metal ion on the electronic and spin properties at the interface. We begin by examining the momentum-integrated valence band spectra of both the pristine Fe(001)-p(1 × 1)O surface and the FePc/Fe(001)-p(1 × 1)O interface, as shown in Figure 1a. In particular, measurements on the pristine surface reveal its distinct electronic structure, where the spectral features near the Fermi level (E_F_) are attributed to antibonding states arising from hybridization between O 2p and Fe 3d orbitals, with a dominant contribution from the latter.^28,29^

**Figure 1:**
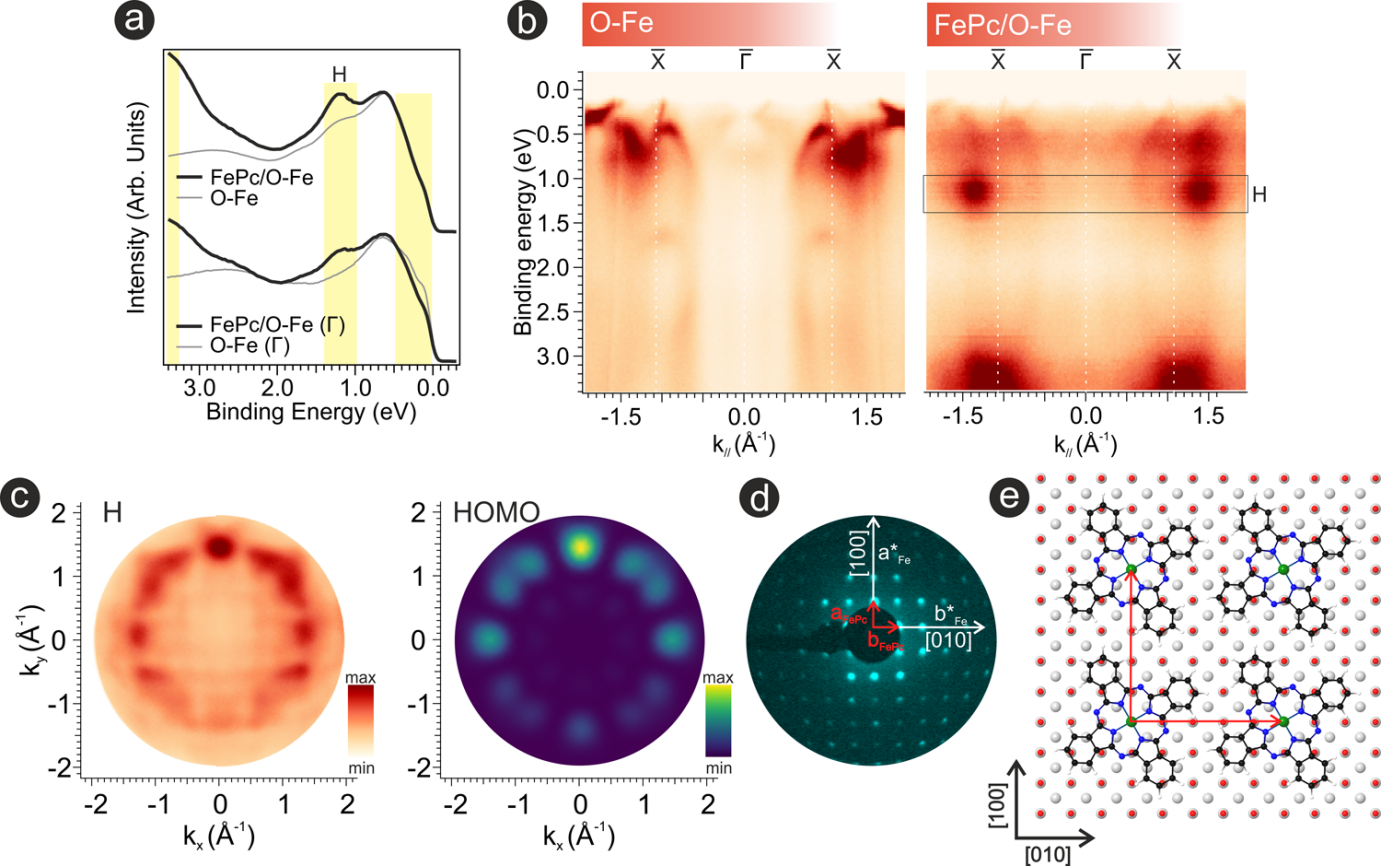
(a) Angle-integrated valence band spectra of the Fe(001)-p(1 × 1)O interface before (gray curves) and after the deposition of an FePc monolayer (black curves). Two integration intervals are shown: [−2, +2] Å^–1^ (top curves) and [−0.7, +0.7] Å^–1^ (labeled Γ, bottom curves). Changes induced by molecular adsorption are highlighted in yellow. (b) Corresponding band maps measured along the X̅−Γ̅−X̅ direction of the substrate’s first Brillouin zone. The black box marks the location of the highest occupied molecular orbital (HOMO, labeled H). (c) Experimental two-dimensional momentum map acquired at 1.2 eV binding energy (H) for the FePc/Fe(001)-p(1 × 1)O interface, compared with the map computed from the HOMO state of the FePc molecule. (d) Low-energy electron diffraction (LEED) pattern of the FePc/Fe(001)-p­(1 × 1)O interface recorded at 20 eV kinetic energy. Substrate and molecular reciprocal vectors are represented by white and red arrows, respectively. (e) Top-view ball-and-stick representation of the FePc adsorption geometry on the Fe(001)-p(1 × 1)O surface, with the main crystallographic directions of the substrate indicated. Atom color code: substrate Fe atoms (gray), oxygen (red), carbon (black), hydrogen (white), nitrogen (blue), and central Fe ion in the phthalocyanine molecule (green).

Following the deposition of FePc, the valence band spectrum exhibits several changes arising from the presence of the molecular overlayer, as highlighted in yellow in Figure 1a. Notably, there is a suppression of the signal near E_F_, the appearance of a distinct peak centered around 1.2 eV binding energy (BE), and an additional broad feature at higher binding energies, *i.e.*, above 2.5 eV. The near-E_F_ variations could be attributed to the formation of HISs, in line with previous reports.^16,30^ The peak at 1.2 eV BE corresponds to the highest occupied molecular orbital (HOMO) of FePc, while the broader structure at higher binding energies is associated with deeper-lying occupied molecular states.^31,32^

The molecular-induced changes are further evident in the band maps measured along the X̅−Γ̅−X̅ direction of the substrate’s first Brillouin zone, shown in Figure 1b. For the pristine Fe(001)-p(1 × 1)O system, the spectral intensity is dominated by sharp, dispersive bands originating from Fe 3d–O 2p hybrid states, which dominate the region near the Fermi level. Upon deposition of the FePc overlayer, these substrate-related features become significantly attenuated across the entire *k*_//_ range, reflecting the damping of substrate photoemission due to screening by the molecular layer. Concurrently, new spectral features emerge that are absent in the pristine system. These appear predominantly at higher *k*_//_ values, particularly beyond 1.2 Å^–1^, and are nondispersive in nature, consistent with emissions from localized molecular orbitals.^31,33,34^

To probe the origin of these features in more detail, we acquired momentum-resolved photoemission maps for both the pristine Fe(001)-p(1 × 1)O substrate and the FePc/Fe(001)-p(1 × 1)O interface, focusing on energy windows near E_F_ and 1.2 eV BE. These molecular signatures provide access to the orbital structure of the adsorbed FePc and can be interpreted using the framework of photoemission tomography (PT).^33,35,36^ In this approach, the measured momentum distribution of the photoelectrons is compared with simulations based on the Fourier transform of density functional theory (DFT)-calculated molecular orbitals (MO),^37^ enabling assignment of features to specific MOs and determination of the molecular on-surface azimuthal orientation.

The momentum map recorded at 1.2 eV BE for the FePc/Fe(001)-p(1 × 1)O interface reveals a clear molecular fingerprint (Figure 1c), while also retaining faint traces of the substrate signal. A direct comparison with the calculated Fourier transform of the FePc HOMO, accounting for rotational domains due to the 4-fold symmetry of the Fe(001) substrate, shows excellent agreement with the experimental data. This comparison allows an unambiguous identification of the spectral features as originating from the HOMO, which possesses *a*_1u_ symmetry. Furthermore, the molecular layer is found to be highly ordered, with molecules exhibiting an azimuthal angle of 29 ± 3° mirrored with respect to the main crystallographic directions of the substrate.

The highly ordered arrangement of FePc on Fe(001)-p(1 × 1)­O is also confirmed by the structural data. Low-energy electron diffraction (LEED) patterns (Figure 1e) reveal the formation of a commensurate (5 × 5) superstructure, with the molecular supercell aligned along the [100] and [010] directions of the substrate (see Figure 1e for a schematic representation of the molecular adsorption).

While the feature observed at 1.2 eV binding energy can be directly attributed to the HOMO of FePc, the spectral intensity detected close to the Fermi level arises from a different mechanism. Rather than originating from a molecular state, this feature is a manifestation of the ordered molecular lattice formed by the FePc overlayer on the Fe(001)-p(1 × 1)O substrate. Specifically, it results from a surface Umklapp scattering process, in which photoelectrons emitted from the substrate bands are elastically scattered by the periodic potential imposed by the adsorbed molecular superstructure.^18–21^ This coherent scattering leads to the appearance of replicated substrate band features in momentum space, shifted by reciprocal lattice vectors of the molecular overlayer. The presence of a well-defined (5 × 5) molecular lattice, confirmed by LEED, provides the structural periodicity necessary for such diffraction to occur. Unlike the localized molecular orbitals, these Umklapp replicas reflect the long-range structural order of the interface rather than the intrinsic electronic states of the molecule.

Figure 2 presents photon energy-dependent two-dimensional momentum maps acquired at 150 meV binding energy for both the bare Fe(001)-p(1 × 1)O substrate (top row) and the FePc/Fe(001)-p(1 × 1)O interface (bottom row), using p-polarized light in the 25–40 eV range. Across all photon energies, the characteristic features of the pristine substrate, originating from the Fe 3d–O 2p states,^29^ are clearly resolved and outline the first surface Brillouin zone (SBZ). Upon FePc deposition, additional features emerge due to Umklapp scattering, forming a superlattice in reciprocal space that reflects the (5 × 5) periodicity of the molecular overlayer. Notably, Umklapp periodicity is consistently observed across the entire photon energy range and leads to the appearance of substrate band replicas that follow the reduced surface Brillouin zone defined by the superstructure. These Umklapp features are more prominently visible at lower photon energies, in agreement with previous reports of Umklapp scattering induced by periodic molecular lattices.^19–21^ In the present case, they appear most distinctly at 30 eV using p-polarized light (see Figure S1 in Supporting Information for comparison between the momentum maps acquired at 30 eV photon energy using p- and s-polarized light).

**Figure 2:**
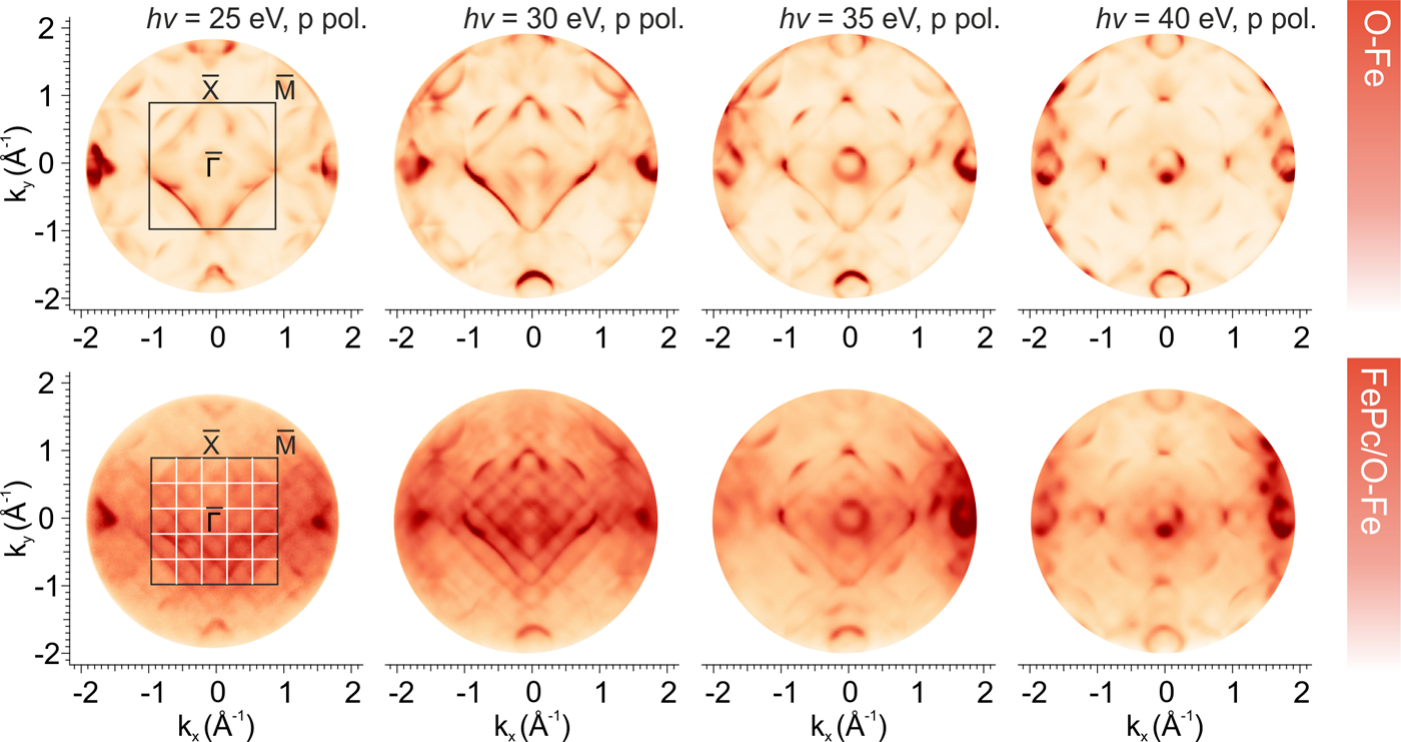
Two-dimensional momentum maps acquired at 150 meV binding energy using p-polarized light in the photon energy range from 25 to 40 eV. The top row shows the maps of the bare Fe(001)-p(1 × 1)O interface, while the bottom row displays the FePc/Fe(001)-p(1 × 1)O interface. In the first map of each row, the contours of the first surface Brillouin zone, along with the main SBZ points, are indicated. For the FePc/Fe(001)-p(1 × 1)O interface, the reduced SBZ is outlined with white lines.

The role of the central metal ion in the diffraction process was further examined through spin-integrated measurements of metal-free H_2_Pc adsorbed onto the same Fe(001)-p(1 × 1)O substrate (Figure S2, Supporting Information). The corresponding LEED pattern confirms the formation of a comparable (5 × 5) superstructure, albeit with slightly less defined diffraction spots. Nevertheless, momentum maps measured near E_F_ reveal an almost identical Umklapp pattern, strongly supporting the conclusion that the observed replicas arise from elastic scattering of substrate photoelectrons by the periodic molecular lattice rather than from hybridization effects associated with the Fe center.

A detailed assessment of the spin character of these features and their behavior at the interface was conducted by using spin-resolved momentum microscopy. This technique provides simultaneous access to the spin and momentum distributions of photoemitted electrons across the whole surface Brillouin zone, offering direct insight into the spin-resolved contributions from both the substrate and the molecular layer.

We first characterize the spin-resolved momentum distribution of the pristine Fe(001)-p(1 × 1)O substrate near the Fermi level (Figure 3). As expected, this surface exhibits well-defined spin-polarized bands, where electronic states are predominantly either spin-up (majority) or spin-down (minority), aligned with the magnetization direction of the substrate.^29^ For all spin-resolved measurements, the samples were magnetized *in situ*, and the data were acquired under remanent magnetization. The spin-resolved momentum map of the substrate reveals that states near the center of the surface Brillouin zone, as well as those forming square-like features with corners at the X̅ point, carry predominantly minority spin character (blue), while features near the M̅-point, associated with O 2p-Fe 3d hybrid bands, show strong majority spin polarization (red), in agreement with previous works.^29^

**Figure 3:**
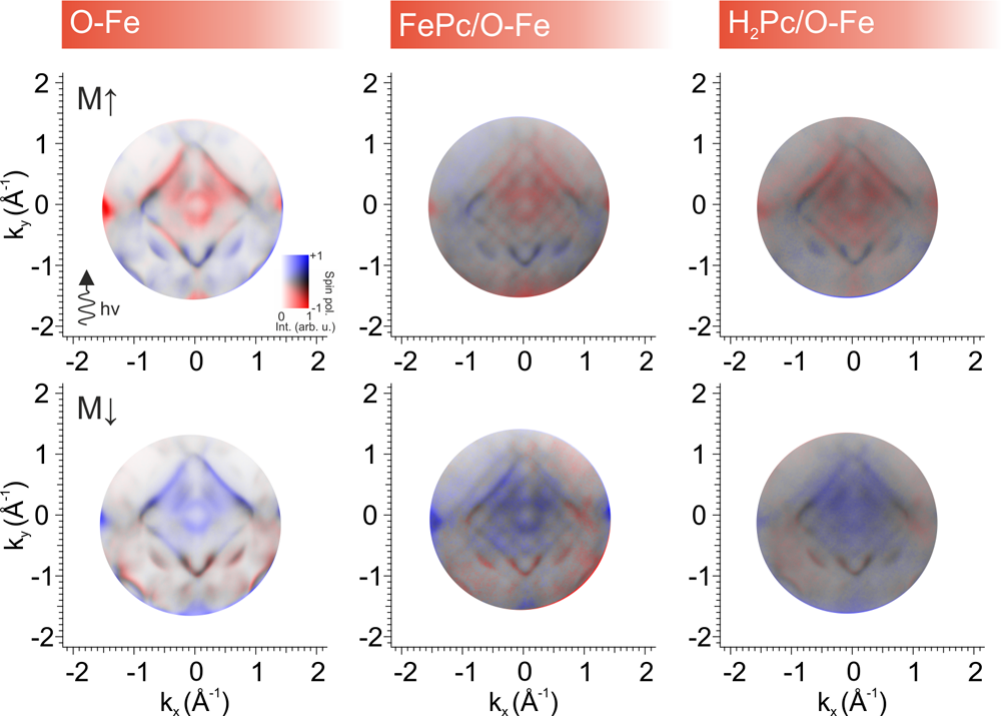
Spin-resolved two-dimensional momentum maps acquired at 150 meV binding energy for the bare Fe(001)-p(1 × 1)­O, the FePc/Fe(001)-p(1 × 1)­O, and the H_2_Pc/Fe­(001)-p­(1 × 1)O interfaces, respectively. Samples were magnetized *in situ* along (top row) and opposite (bottom row) to the beam propagation direction and measured in remanence.

We then investigated the spin character of the scattering-induced features at the FePc/Fe(001)-p(1 × 1)O interface. The spin-resolved momentum map at 150 meV BE confirms that the primary spin-polarized substrate bands remain clearly discernible through the molecular layer. More importantly, their replicas, arising from the surface Umklapp process, retain the original spin polarization of the substrate bands. This behavior is further validated by reversing the magnetization direction (M↑ and M↓ along the *y*-axis), which results in a full reversal of the spin polarization signal, as expected for a ferromagnetic substrate with a low coercive field.

Consistent with the idea that the spin polarization in the Umklapp process originates from the substrate bands, the same behavior is also observed for the metal-free H_2_Pc overlayer on Fe(001)-p(1 × 1)­O. The spin-resolved momentum map acquired at 150 meV binding energy reveals that the substrate’s spin-polarized bands remain visible beneath the molecular layer, and their scattering-induced replicas likewise retain the spin polarization of the underlying states. While the overall spin-resolved band structure closely mirrors that of the FePc interface, the Umklapp features appear to be broader in momentum space. This reduced clarity correlates with the slightly more diffuse LEED pattern observed for H_2_Pc in Figure S2 of the Supporting Information, suggesting a lower degree of long-range structural order in the molecular lattice compared to FePc.

At the same time, the molecular overlayer induces an overall suppression of the substrate spectral features, reflecting both the attenuation of photoelectrons due to the presence of the molecular film and the partial quenching of substrate-related intensity at the interface. This effect is clearly illustrated in Figure 4, which presents spin-resolved band maps measured along the X̅−Γ̅–M̅ direction of the surface Brillouin zone for the different interfaces investigated. For both FePc and H_2_Pc overlayers, the intensity of the pristine Fe(001)-p(1 × 1)O bands is significantly reduced compared with the bare substrate, while the associated Umklapp replicas remain visible and retain the original spin polarization.

**Figure 4:**
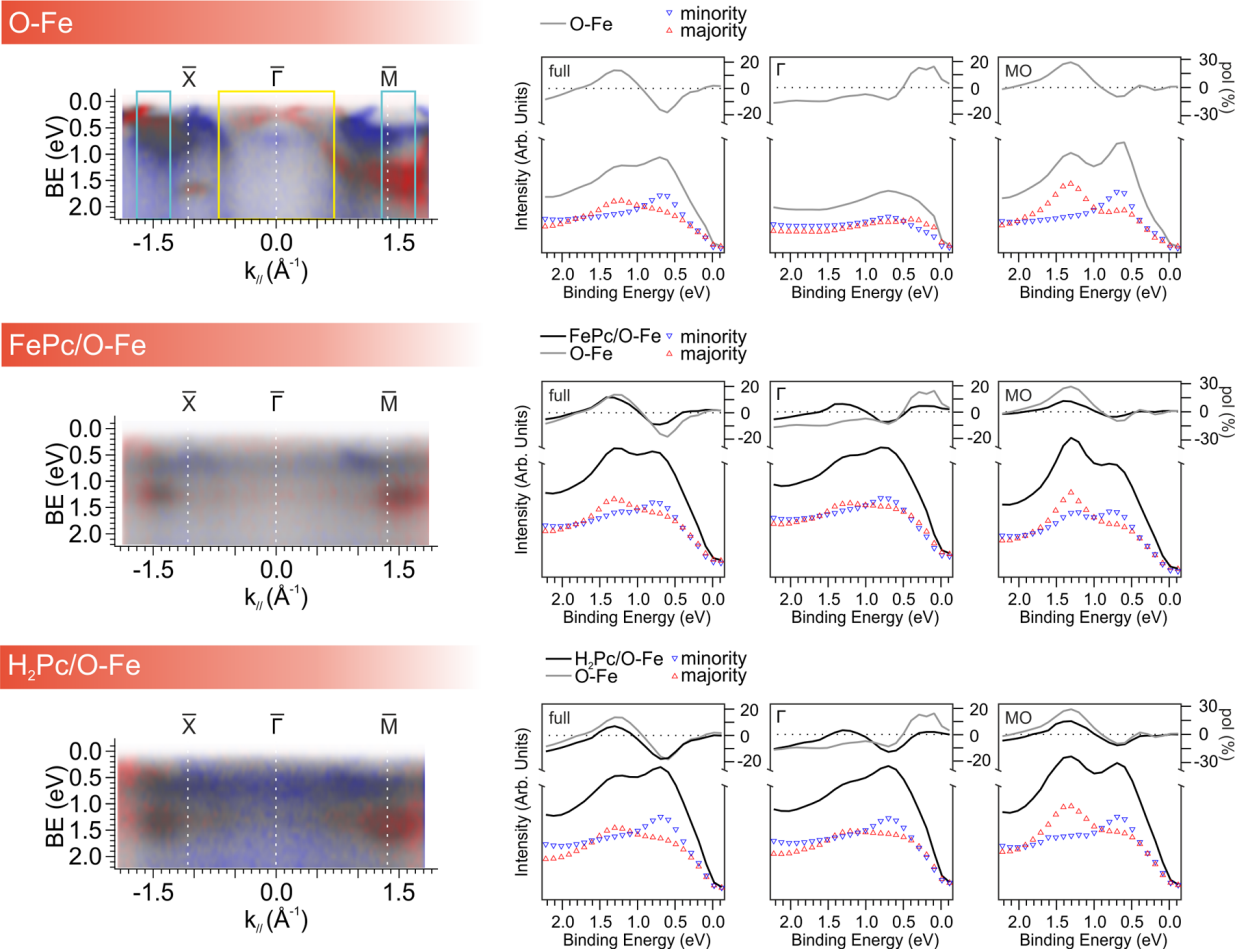
Spin-resolved band maps measured along the X̅−Γ̅–M̅ direction of the substrate’s first Brillouin zone for the bare Fe(001)-p(1 × 1)­O, the FePc/Fe(001)-p(1 × 1)­O, and the H_2_Pc/Fe­(001)-p­(1 × 1)O interfaces (left column). Corresponding majority- and minority-spin-resolved spectra measured for the three interfaces are shown in the right column. Different momentum integration intervals, corresponding to different acceptance angles, are plotted (full range: [−1.8, +1.8] Å^–1^, Γ range: [−0.7, +0.7] Å^–1^ (yellow box), and MO range around the molecular features (cyan box)). Reference boxes are indicated in the top-left panel. Spin polarization curves are displayed in the top part of each panel. Measurements are performed under remanent magnetization after applying a saturating field (M↑).

To gain a more quantitative understanding of how molecular adsorption affects spin polarization at the spinterface, we analyzed spin-resolved energy distribution curves extracted from different regions of momentum space, as shown in the right panel of Figure 4. These spectra were acquired for both FePc/Fe(001)-p(1 × 1)O and H_2_Pc/Fe­(001)-p­(1 × 1)O interfaces and integrated over three distinct momentum intervals: the full momentum range (−1.8 to +1.8 Å^–1^), a narrower window centered around normal emission (Γ: – 0.7 to +0.7 Å^–1^), and a region localized around the MO features. The corresponding spin polarization curves are displayed in the upper part of each panel, enabling a direct comparison of spin-resolved behavior across different areas of reciprocal space.

The full-range integration reflects a broad angular acceptance, while the Γ̅-centered integration isolates contributions close to normal emission, where the Umklapp features are most pronounced. This distinction is especially relevant for spin-resolved measurements using angle-integrating techniques, where the analyzer’s limited acceptance angle primarily samples the region around the Γ̅ point, making the measurement particularly sensitive to Umklapp-induced features. In contrast, the MO region targets momentum space dominated by molecular states, allowing us to specifically assess their contribution to spin polarization.

From this analysis, several significant trends emerge. Over the full angular range, molecular adsorption leads to a clear suppression of overall spin polarization for both FePc and H_2_Pc. The observed suppression of spin polarization upon FePc and H_2_Pc adsorption is consistent with a spin-independent depolarization mechanism, as proposed by Greber *et al.* for ordered, nonmagnetic overlayers.^22^ The periodic potential introduced by the molecular lattice facilitates surface Umklapp scattering of substrate-derived electrons, leading to a momentum-space redistribution of the spectral weight and altered photoemission matrix elements. Crucially, although the scattering itself is spin-independent, the available phase space for electron–hole excitations differs for spin-up and spin-down electrons due to the spin-polarized band structure of the substrate. This imbalance results in an effective depolarization of the emitted photoelectrons, even in the absence of direct hybridization between molecular and substrate states, underscoring the nontrivial impact of molecular ordering on spin-resolved electronic structure at organic/ferromagnetic interfaces.

Overlayer-induced changes appear even more evident when focusing on the Γ̅-centered region, where the Umklapp replicas are most intense. The spin polarization near the Fermi level, as associated with the substrate bands scattered by the molecular lattice, is suppressed due to overlap with Umklapp scattered bands of the opposite spin. Conversely, the feature at around 1.2 eV binding energy, attributed to the HOMO of FePc, exhibits a reversal of the spin polarization near the Γ̅ point. This can be attributed to the spectral overlap with spin-polarized substrate bands that are backscattered into this region.

When examining spin-resolved spectra integrated around the MO region, the polarization curves closely resemble those obtained from the full angular range, even with a reduced intensity. This similarity suggests that the changes observed near normal emission are not primarily due to the intrinsic spin character of the molecular states but rather stem from Umklapp scattering processes that also redistribute substrate-derived photoelectrons at binding energies at which the molecular states are present.

Moreover, we highlight that the presence of Umklapp scattering can be clearly observed in the spin-resolved band maps (Figure 4, left) as nondispersing bands over the entire first Brillouin zone. This is consistent with the backfolding of the most intense features of minority spin (at a binding energy of 0.6 eV) and majority (at a binding energy of 1.3 eV) spin bands of the substrate due to the presence of the ordered organic overlayer.

Overall, this analysis underscores the importance of disentangling molecular electronic contributions from diffraction-induced effects when interpreting spin polarization at complex organic–ferromagnetic interfaces and suggests that structural ordering at the interface can strongly modulate, and even obscure, the true spin character of buried states.

In conclusion, this study demonstrates the critical role of structural order in governing the spin-resolved electronic properties of spinterfaces. Using spin- and momentum-resolved photoemission spectroscopy combined with photoemission tomography, we systematically investigated FePc and H_2_Pc monolayers adsorbed on the Fe(001)-p(1 × 1)O substrate. Our results show that while the HOMO of FePc gives rise to distinct molecular emission, the spectral intensity observed near the Fermi level originates from surface Umklapp scattering, an elastic diffraction mechanism enabled by the long-range order of the molecular overlayer. Crucially, these diffraction-induced replicas inherit the spin polarization of the underlying ferromagnetic substrate, regardless of the magnetic nature of the molecular species. A detailed momentum-resolved spin analysis further confirms that spin polarization near normal emission is predominantly governed by scattering rather than direct contributions from spin-polarized molecular orbitals. Overall, these findings highlight the intricate interplay between molecular ordering, spin polarization, and electron diffraction at organic/ferromagnetic interfaces, establishing structurally defined spinterfaces as a powerful model platform for disentangling spin interactions at hybrid interfaces with direct implications for the design and characterization of spin-functional materials and devices.

## Supplementary Material



## Notes

Sample preparation and methods; additional polarization-dependent momentum maps; LEED and ARPES of the H_2_Pc/Fe­(001)-p­(1 × 1)O interface; spin-resolved band maps measured along X̅−Γ̅–X̅ and M̅−Γ̅–M̅ for the bare Fe(001)-p(1 × 1)­O, the FePc/Fe(001)-p(1 × 1)­O, and the H_2_Pc/Fe­(001)-p­(1 × 1)O interfaces; spin-resolved spectra measured with reversed magnetization (PDF)

## †

Physical and Computational Sciences Directorate and Institute for Integrated Catalysis, Pacific Northwest National Laboratory, Richland, Washington 99354, USA
