# Supplementary material for: Spin and Momentum Mapping of Highly Oriented Spinterfaces
Source: Nano Lett. 2025 Nov 28;25(49):17138–44. doi: 10.1021/acs.nanolett.5c04710 (PMC12874631; doi:10.1021/acs.nanolett.5c04710)
Supplement: Supplementary file 1 [file nl5c04710_si_001.pdf]

SUPPORTING INFORMATION

Spin and momentum mapping of highly oriented spinterfaces

Iulia Cojocariu,[1,2,3] Daniel Baranowski,[3,4] Vitaliy Feyer,[3,5] Matteo Jugovac,[1,2] and Claus Michael Schneider[3,5,6]*

1 Physics Department, University of Trieste, 34127 Trieste, Italy

2 Elettra Sincrotrone Trieste S.C.p.A., 34149 Basovizza, Trieste, Italy

3 Peter Grünberg Institute (PGI-6), Forschungszentrum Jülich GmbH, 52425 Jülich, Germany

4 Present address: Physical and Computational Sciences Directorate and Institute for Integrated Catalysis, Pacific Northwest National Laboratory, Richland, Washington 99354, USA

5 Faculty of Physics and Center for Nanointegration Duisburg-Essen (CENIDE), University of Duisburg-Essen, 47048 Duisburg, Germany

6 Department of Physics and Astronomy, UC Davis, Davis CA 95616, USA

**Corresponding author: Iulia Cojocariu, email: iulia.cojocariu@units.it*

Experimental Section

Sample preparation. The iron film was prepared by initially cleaning a MgO(100) substrate under vacuum through two cycles of argon ion sputtering (2 keV Ar^+) followed by annealing at 870 K for 45 minutes.[1] Subsequently, a 300 nm-thick Fe(100) layer was epitaxially deposited *in situ* onto the clean MgO using electron-beam evaporation from Fe solid rod. This growth method favors an in-plane magnetization along the Fe [100] direction. Surface cleaning of the Fe film in the experimental setup was performed by alternating sputtering with 0.5 keV Ar^+ ions and thermal annealing up to 870 K. To generate the Fe(001)-p(1×1)O phase, the iron surface was exposed to 30 L of molecular oxygen at a sample temperature of around 820 K. After the gas dose, the sample was annealed at 870 K for 5 minutes to remove excess oxygen and promote the formation of the desired surface reconstruction. The FePc and H_2Pc molecules were thermally sublimated at 700 and 570 K, respectively, from a homemade Knudsen cell type evaporator onto the substrate kept at room temperature, up to monolayer coverage. Coverage calibration was performed on a Ag(110) crystal and verified by valence band spectroscopy, exploiting the well-known appearance of an additional spectral feature associated with the second molecular layer, which is electronically decoupled from the metallic substrate.[2]

Methods. Spin-integrated and spin-resolved momentum microscopy measurements were conducted at the NanoESCA beamline of the Elettra synchrotron facility in Trieste, Italy. The experiments utilized a photoemission electron microscope operating in k-space mode (k-PEEM).[3] The samples were illuminated with soft X-rays delivered by two Apple-II type undulators, which provide a tunable photon energy range between 25 and 1300 eV and support various polarization modes, including linear (horizontal and vertical) and elliptical. In the specific geometry of the k-PEEM setup at the NanoESCA beamline (65° incidence angle with respect to the surface normal), p-polarized light (electric field parallel to the plane of incidence) corresponds to vertical

polarization, while s-polarized light (electric field perpendicular to the plane of incidence) corresponds to horizontal polarization. Control over the kinetic energy of emitted photoelectrons was achieved by adjusting the sample bias voltage. The emitted electrons were collected via an optical column, passed through an energy filter in a double-hemispherical analyzer configuration (IDEA), and projected onto a two-dimensional detector. For spin resolution, the system includes a W(001) spin filter within the optical column, enabling imaging in both real and reciprocal space with in-plane spin sensitivity. The degree of spin polarization was calculated assuming constant spin sensitivity values of 0.42 and 0.05, depending on the scattering energy used.[4] As a result, the spin polarization P can vary from −100% (fully spin-down) to +100% (fully spin-up). Before spin-resolved measurements, the sample was magnetized *in situ* with an external magnetic field of approximately 0.5 T to align the Fe magnetic domains along the easy magnetization axis ([001]). All measurements were carried out with the sample maintained at liquid nitrogen temperature. Unless otherwise specified, electronic structure mapping was performed using p-polarized light at a photon energy of 30 eV.

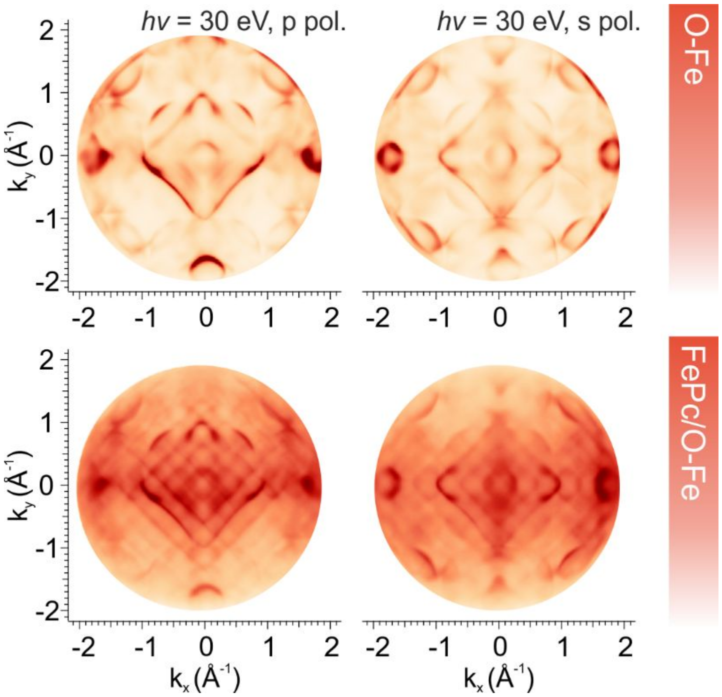

Figure S1. Two-dimensional momentum maps acquired at 150 meV binding energy using p- and s-polarized light at a photon energy of 30 eV. Top row reproduces the maps of the bare Fe(001)-p(1x1)O interface, while the bottom row reproduces the FePc/ Fe(001)-p(1x1)O interface.

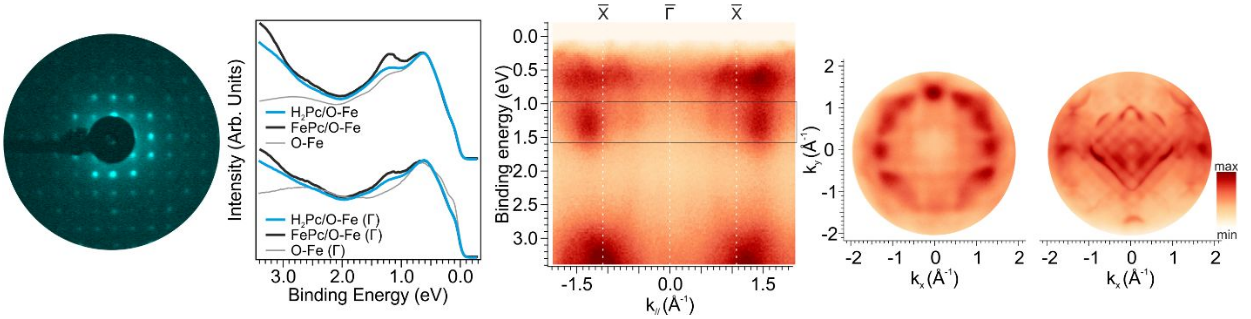

Figure S2. **a)** Low energy electron diffraction pattern of the H_2Pc/Fe(001)-(1x1)O interface acquired at 20 eV kinetic energy. **b)** Angle-integrated valence band spectra of the Fe(001)-(1x1)O interface before (gray curves) and after the deposition of a FePc monolayer (black curve) and H_2Pc monolayer (light blue curve), respectively. Two integration intervals are represented, [-2,+2] $Å^{-1}$ (top curves) and [-0.7,+0.7] $Å^{-1}$ (labelled as Γ, bottom curves). **c)** Corresponding band maps measured along the X-Γ-X direction of the substrate first Brillouin Zone. Black box indicates the location of the highest occupied molecular orbital (HOMO). **d)** Experimental two-dimensional momentum map acquired at 1.2 eV and at 150 meV binding energy for the H_2Pc/Fe(001)-(1x1)O interface.

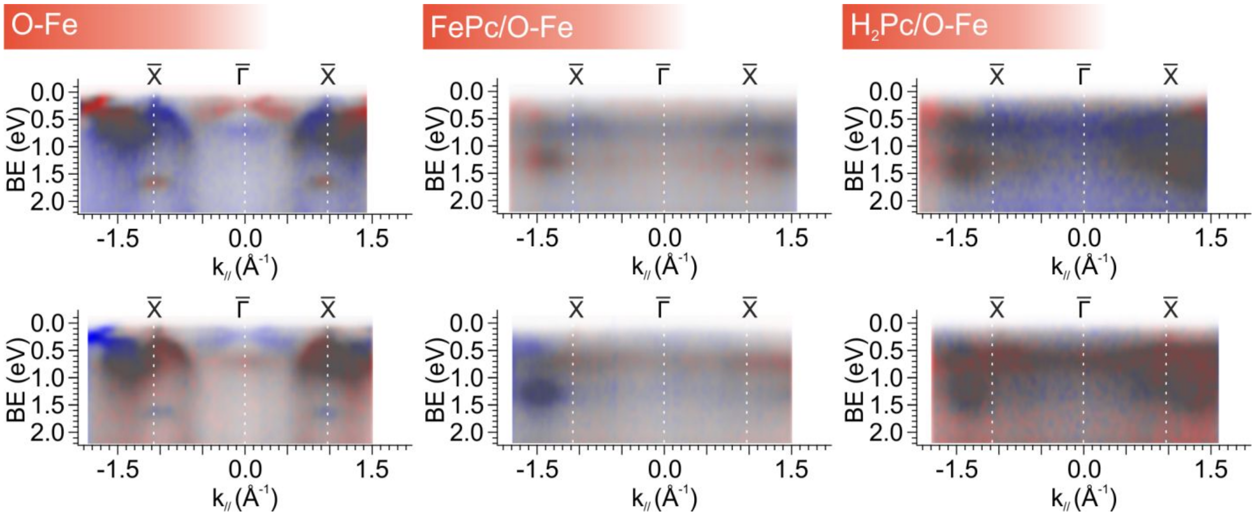

Figure S3. Spin-resolved band maps measured along the X-Γ-X direction of the substrate first Brillouin Zone for the bare Fe(001)-p(1x1)O, the FePc/Fe(001)-p(1x1)O and the H_2Pc/Fe(001)-p(1x1)O interfaces, respectively.

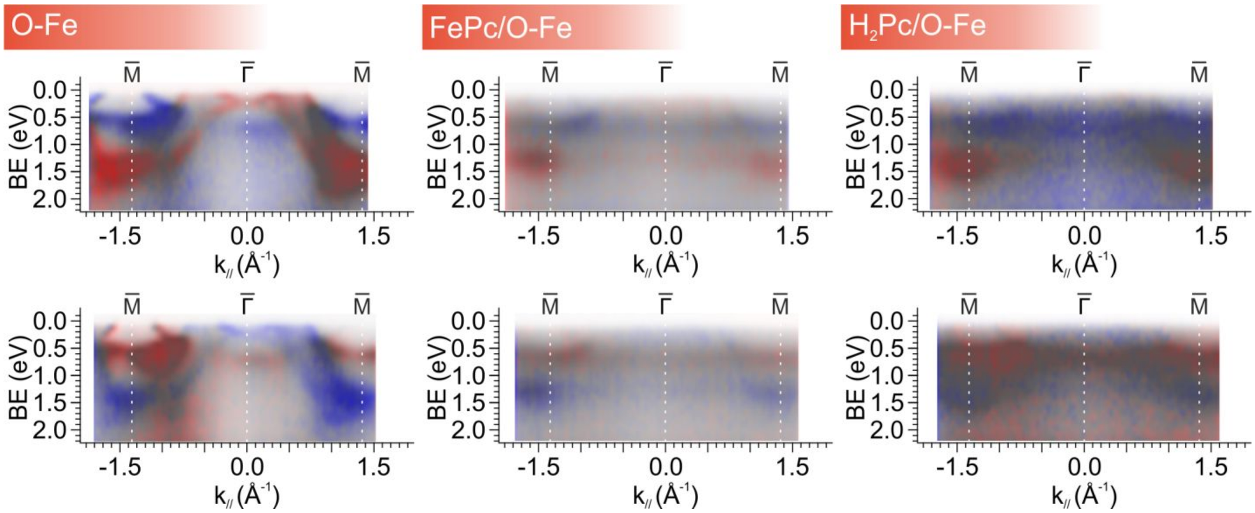

Figure S4. Spin-resolved band maps measured along the M-Γ-M direction of the substrate first Brillouin Zone for the bare Fe(001)-p(1x1)O, the FePc/Fe(001)-p(1x1)O and the H_2Pc/Fe(001)-p(1x1)O interfaces, respectively.

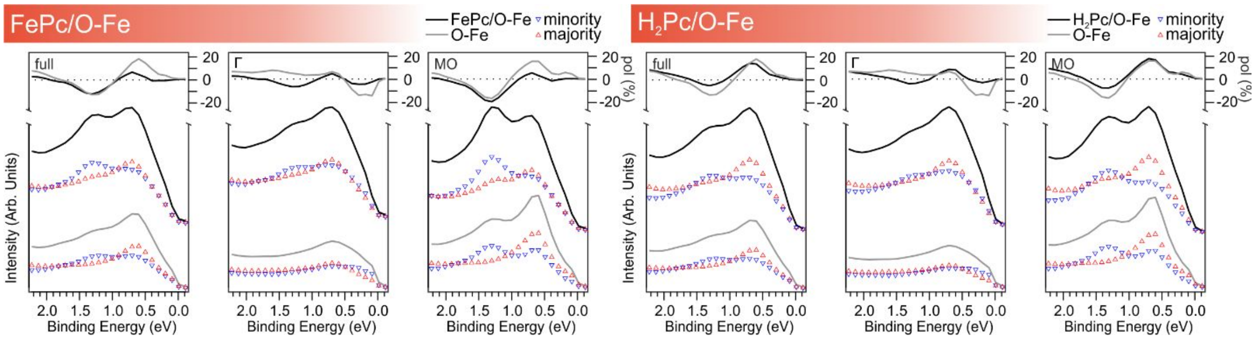

Figure S5. Majority and minority spin-resolved spectra measured for the FePc/Fe(001)-p(1x1)O and H_2Pc/Fe(001)-p(1x1)O interfaces. Different integration intervals, corresponding to different acceptance angles, are plotted (full: [-1.8;+1.8] $Å^{-1}$, Γ: [-0.7,+0.7] $Å^{-1}$ and MO: around the molecular features). Spin polarization curves are reported in the top part of each panel. Measurements are performed in remanence ($M_{\downarrow}$).

References

(1) Janas, D. M.; Droghetti, A.; Ponzoni, S.; Cojocariu, I.; Jugovac, M.; Feyer, V.; Radonjić, M. M.; Rungger, I.; Chioncel, L.; Zamborlini, G.; Cinchetti, M. Enhancing Electron Correlation at a 3d Ferromagnetic Surface. *Advanced Materials* **2023**, *35* (3), 2205698. https://doi.org/10.1002/adma.202205698.

(2) Cojocariu, I.; Jugovac, M.; Sarwar, S.; Rawson, J.; Sanz, S.; Kögerler, P.; Feyer, V.; Schneider, C. M. Semiconductor Halogenation in Molecular Highly-Oriented Layered p–n (n–p) Junctions. *Adv Funct Materials* **2022**, *32* (51), 2208507. https://doi.org/10.1002/adfm.202208507.

(3) Wiemann, C.; Patt, M.; Krug, I. P.; Weber, N. B.; Escher, M.; Merkel, M.; Schneider, C. M. A New Nanospectroscopy Tool with Synchrotron Radiation: NanoESCA@Elettra. *e-J. Surf. Sci. Nanotechnol.* **2011**, *9*, 395–399. https://doi.org/10.1380/ejssnt.2011.395.

(4) Tusche, C.; Ellguth, M.; Krasyuk, A.; Winkelmann, A.; Kutnyakhov, D.; Lushchyk, P.; Medjanik, K.; Schönhense, G.; Kirschner, J. Quantitative Spin Polarization Analysis in Photoelectron Emission Microscopy with an Imaging Spin Filter. *Ultramicroscopy* **2013**, *130*, 70–76. https://doi.org/10.1016/j.ultramic.2013.02.022.
